# Triple Infectious Syndrome: Pulmonary Tuberculosis and HIV in a Case of Pure Neuritic Leprosy With Underlying Diabetes Mellitus

**DOI:** 10.7759/cureus.66105

**Published:** 2024-08-04

**Authors:** Mohanakrishnan Deivasigamani, Dileepan Sureshgraham, Sruthi Priyadarsini Srikanth, Balamurugan Santhalingam, Chandrasekar Chokalingam

**Affiliations:** 1 Respiratory Medicine, ACS Medical College and Hospital, Chennai, IND

**Keywords:** type 2 diabetes mellites, national aids control programme, national leprosy eradication programme, national tb elimination program, dolutegravir, rifampicin, hiv, pulmonary tuberculosis, pure neuritic leprosy

## Abstract

Pulmonary tuberculosis (PTB), human immunodeficiency virus (HIV), and leprosy are of public health importance, as all three diseases are communicable and contribute to disease burden in society. Co-infection with these three entities is extremely rare but leads to significant mortality and morbidity. We report a case that highlights the diagnostic challenges and therapeutic management of a patient who was diagnosed with pure neuritic leprosy on multibacillary-multidrug therapy (MB-MDT) and subsequently co-diagnosed with PTB and HIV. The patient was started on anti-tubercular therapy and anti-retroviral therapy for treatment under India’s national health programs, which play a major role in treating those of low socioeconomic status. The optimization of these therapeutic drugs is quite challenging during treatment due to potential drug interactions and toxicities. High clinical suspicion is required to rule out PTB before initiating rifampicin-containing MB-MDT, which can lead to rifampicin-resistant TB and screening for HIV. As there is a social stigma associated with these patients, they require good psychological support during and after treatment.

## Introduction

Pure neuritic leprosy (PNL), accounting for 4%-18% of leprosy cases, is prevalent in South India, where 18% of new cases are reported [1]. The Indian Association of Leprologists recognized PNL as a distinct type of leprosy, including it in their Indian classification system in 1982. With the introduction of multidrug therapy (MDT) in 1982, the World Health Organization simplified the classification of leprosy into paucibacillary and multibacillary categories for therapeutic purposes, which was followed under India’s National Leprosy Eradication Programme (NLEP). Tuberculosis (TB) and leprosy are chronic granulomatous diseases caused by *Mycobacterium tuberculosis* and *Mycobacterium leprae*, respectively. There have been concerns about drug resistance in TB treatment in cases in which leprosy is diagnosed before TB and rifampicin is given once a month. Those living with the human immunodeficiency virus (HIV) are 18 times more likely to develop active TB. In India, 71,000 cases of TB were reported among those living with HIV in 2019 [2]. Those with diabetes mellitus (DM) are also two to four times more likely to develop TB, with 30% of those with TB also having DM [3]. There have been case reports of pulmonary tuberculosis (PTB) and lepromatous leprosy in a patient with HIV [4] and reactivation of cutaneous TB and leprosy in a patient with HIV [5]. We report a case of co-diagnosed HIV and PTB in a case of PNL with type 2 DM (T2DM), in which the patient was diagnosed and treated via India’s national health programs.

## Case presentation

**Figure 1:**
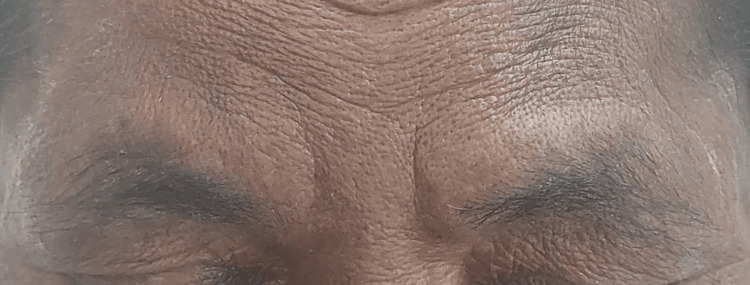
Bilateral madarosis Loss of lateral aspect of eyebrows on both sides

**Figure 2:**
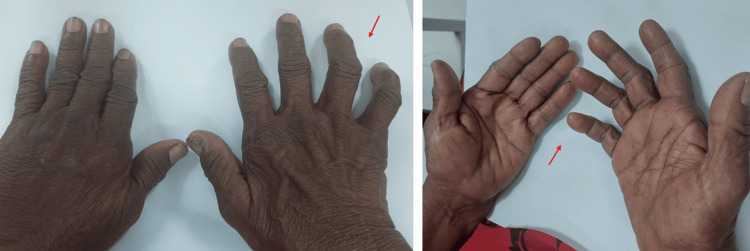
Right partial mobile claw hand Pure neuritic leprosy involving the ulnar two digits of the right hand (red arrow)

A woman in her early 50s was first seen in our hospital in June 2023 to assess the conversion of anti-tubercular therapy (ATT) from the intensive phase to the continuous phase. In terms of past medical history, in October 2022, she had complaints of weakness, difficulty in extending her fingers and holding objects, and decreased sensation over her right hand for one year. On clinical examination, bilateral madarosis was discovered, as seen in Figure 1. Touch sensation was decreased over the median nerve distribution, and loss of sensation was noted over the ulnar nerve distribution over the right hand. Peripheral nerve examination showed Grade II thickening and tenderness over the right ulnar nerve and Grade I thickening over the right radial cutaneous nerve without tenderness. Wasting of the thenar and hypothenar muscles was present, and her Medical Research Council grading of muscle power was 4/5. Card and Book tests were positive, and mobile partial claw hand deformity was present over the right hand, as seen in Figure 2. The diminished sensation was present bilaterally over the lower extremities. A slit skin smear was negative for *M. leprae*, acid-fast bacilli, but a lepromin skin test was positive for early reaction (Fernandez reaction), with a red area of 10 x 10 mm. As a nerve conduction study of the right ulnar nerve showed axonal polyneuropathy, she was advised for nerve biopsy, which she was not willing to complete due to the invasiveness of the procedure. She was diagnosed with PNL/type 1 Lepra reaction/right partial mobile claw hand and started on multibacillary-multidrug therapy (MB-MDT) under the NLEP with oral prednisolone 40 mg once daily and regular physiotherapy, as seen in Table 1. The patient received follow-up once every two weeks, and the oral prednisolone dose was tapered gradually as per NLEP. The patient was diagnosed with T2DM four years prior and was on metformin and glimepiride with good control. The patient’s symptoms improved, and her glycemic level, blood count, liver, and renal parameters were within normal limits during follow-ups.

**Table 1:** Drug regimen for pure neuritic leprosy, pulmonary tuberculosis, and HIV MB-MDT: multibacillary-multi drug therapy; ATT: anti-tubercular therapy; IP: intensive phase; CP: continuous phase; ART: anti-retroviral therapy; FDC: fixed dose combination

Regimen	Drugs	Dose	Frequency	Duration
MB-MDT	Rifampicin(R)/Clofazimine(Cfz)	600 mg/300 mg	Once monthly	12 months
MB-MDT	Dapsone(D)/Clofazimine(Cfz)	100 mg/50 mg	Daily once	12 months
ATT-4FDC (IP)	Isoniazid(H)/Rifampicin(R)/Pyrazinamide(Z)/Ethambutol(E)	75/150/400/275 mg	4 pills daily once	2 months
ATT-3FDC (CP)	Isoniazid(H)/Rifampicin(R)/Ethambutol(E)	75/150/275 mg	4 pills daily once	4 months
ART-FDC	Dolutegravir (DTG)/Lamivudine(3TC)/Tenofovir (TDF)	50/300/300 mg	1 pill daily morning	Rest of life
ART-extra	Dolutegravir (DTG)	50 mg	Daily night	Till ATT

In February 2023, the patient returned with complaints of genital itching and whitish discharge for two weeks. As a KOH mount test showed pseudohyphae suggestive of vaginal candidiasis, she was started on fluconazole and asked to return for follow-up after two weeks.

**Figure 3:**
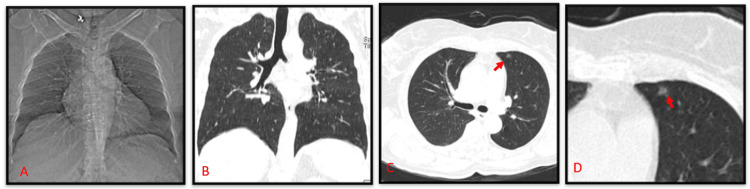
Computed tomography - thorax A) topogram; B) coronal; C and D) axial The red arrow shows a small parenchymal lesion in the superior lingula

In April 2023, the patient still complained of itching and discharge over the genital area. Therefore, she was tested for HIV antibodies, which were reactive. In addition, her CD4 count was 894 cells/mm^3^. Therefore, she planned to start anti-retroviral therapy (ART) after post-test counseling regarding HIV. She had been married twice, now separated, and had one daughter who was healthy with no symptoms. She also complained of occasional cough with scanty mucoid sputum for 15 days and was screened for PTB. Chest X-ray showed no significant abnormalities, and a computed tomography scan of the thorax (Figure 3) showed a small parenchymal lesion in the superior lingula. The patient’s sputum AFB smear was negative, but her Xpert/MTB-RIF was positive for MTB (low), and rifampicin resistance was not detected. Therefore, she was started on ATT (intensive phase) under the National TB Elimination Program (NTEP) (Table 1) based on weight band (58 kg). She was asked to stop monthly rifampicin for leprosy as she would be taking it daily for PTB. She was tolerating ATT well and started on ART under the National AIDS Control Programme (NACP) two weeks after starting ATT with extra dolutegravir 50 mg at night due to rifampicin drug interaction. Her liver and renal functions were monitored and found to be within normal limits.

Upon examination at our hospital in June 2023, bilateral vesicular breath sounds were heard on auscultation of the chest. The patient’s blood parameters were within normal limits, and her sputum AFB smear was negative. Therefore, her ATT regimen was changed to a continuous phase (Table 1). During her second review at the end of September 2023, she was again assessed for stopping ATT. Due to her HIV status and T2DM, she was advised to extend her ATT regimen (continuous phase) for another three months in spite of radiological and bacteriological normalcy owing to the high relapse rate of PTB in such patients.

**Figure 4:**
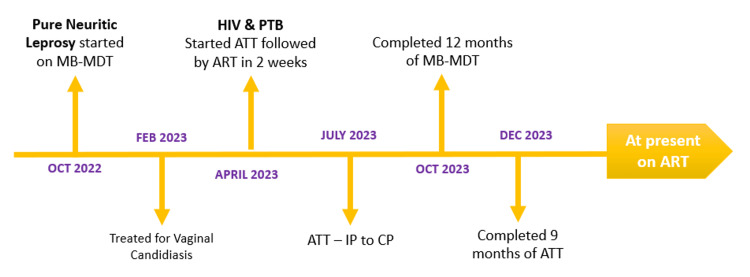
Timeline of the case presentation A chronological record of care from the initial diagnosis of pure neuritic leprosy to the completion of antitubercular therapy and current status HIV: human immunodeficiency virus; PTB: pulmonary tuberculosis; MB-MDT: multibacillary-multi drug therapy; ATT: anti-tubercular therapy; IP: intensive phase; CP: continuous phase; ART: anti-retroviral therapy

At present, the patient has completed nine months of ATT and 12 months of MB-MDT and is continuing with ART. Her extra dolutegravir was stopped after two weeks of completing ATT (continuous phase) as per NACP guidelines. Her condition improved with the alleviation of symptoms and regular physiotherapy. Figure 4 shows the timeline of the case presentation.

## Discussion

Here we reported a rare case of triple infectious disease, highlighting its diagnostic and therapeutic challenges and the need for screening of diseases that can occur concurrently. Such an occurrence is of importance as it can cause severe morbidity and mortality if not intervened at the right time with appropriate treatments, in which some infections are curable.

In this case, when the patient was diagnosed with PNL and started on MB-MDT and oral corticosteroids during the six months before being diagnosed with HIV and PTB, there may have been a possible latent TB infection that led to an active TB infection. Normal chest X-rays and minimal parenchymal lesions on CT-thorax indicated a possible endobronchial spread of TB, which was detected by Xpert MTB-RIF assay. In a study by Yoo et al., 16% of HIV patients exhibited a normal chest X-ray, of which 44% had PTB [6]. In a study by Padyana et al., 9% of patients with HIV-TB co-infection exhibited normal chest X-rays [7]. Similar studies of CT-thorax have shown less cavitation and consolidation in HIV seropositive patients with PTB [8-10] as seen in our case. Leprosy-TB co-infection was reported in patients diagnosed with either borderline or lepromatous leprosy who were on corticosteroids for type 1 lepra reaction [11]. In most leprosy-TB co-infections, TB follows leprosy infection eventually [11,12]. Even though our patient was on rifampicin monthly for leprosy, her Xpert MTB-RIF assay did not detect rifampicin resistance. Unlike TB, the level of host systemic immunosuppression due to HIV infection does not appear to significantly modify the natural course or clinical presentation of leprosy [13]. When the patient was started on ATT, rifampicin was omitted from MDT for leprosy and given in the doses recommended as per NTEP guidelines. Such patients require regular follow-up, as they may develop drug resistance. In spite of all the drugs the patient was on, monitoring of her liver and renal function tests showed that they were within normal limits.

HIV affects T-helper type 1 cell-mediated immunity, which is an important immune response against TB. Our patient was started on ATT prior to ART with a CD4 count of 894 cells/mm^3^ under NACP (DTG/3TC/TDF), with extra dolutegravir 50 mg added at night due to rifampicin interaction, which reduces bioavailability and increases clearance of dolutegravir [2,14]. HIV-TB co-infection is complicated by drug interactions between anti-tubercular drugs and anti-retroviral drugs, additional drug toxicities due to the regimen, TB-associated immune reconstitution inflammatory syndrome, adherence to treatment, and issues of pill burden, which can be reduced by fixed-dose combinations. Cotrimoxazole preventive therapy was not initiated as the patient was already on dapsone for leprosy, and her CD4 count was within normal limits [2].

DM impairs cell-mediated immunity due to poor glycemic control, which affects the cytokine response and alters the defense mechanism in alveolar macrophages, leading to a higher risk of TB infection, and immune deficiency in these patients is sufficient for re-activation of latent TB. Approximately 10% of patients with immunocompetency will experience reactivation of TB in their lifetime, with the risk increasing by 10% every year in patients with immunodeficiency [3]. Throughout the course of treatment, our patient had good glycemic control with oral hypoglycemic agents, and regular monitoring of glycemic levels was advised, as such patients have a high chance of TB reactivation.

## Conclusions

High clinical suspicion of PTB is required before initiating MDT for leprosy to prevent rifampicin-resistant TB and to screen for HIV, as TB can cause high mortality in these patients. As HIV screening is routinely performed for PTB patients, we recommend that all patients with leprosy should also be screened for HIV. Proper referrals at regular intervals to specialists are important for optimizing therapeutic management and preventing mistreatment in these patients. Our patient was treated under India’s national health programs, which led to their compliance with treatment. Furthermore, the pill burden is reduced with fixed-dose combinations. At the completion of MB-MDT and ATT, our patient was happy and satisfied. Such patients also require good psychological support from their family and friends.
